# Human Genome Safe Harbor Sites: A Comprehensive Review of Criteria, Discovery, Features, and Applications

**DOI:** 10.3390/cells15010081

**Published:** 2026-01-04

**Authors:** Amer Ahmed, Daria Di Molfetta, Giorgia Natalia Iaconisi, Antonello Caponio, Ansu Singh, Aasia Bibi, Vincenza Dolce, Luigi Palmieri, Vincenzo Coppola, Giuseppe Fiermonte

**Affiliations:** 1 Department of Biosciences, Biotechnologies and Environment, University of Bari Aldo Moro, 70125 Bari, Italy; aa.biotechiub@gmail.com (A.A.); daria.dimolfetta@uniba.it (D.D.M.); giorgia.iaconisi@uniba.it (G.N.I.); antonello.caponio@gmail.com (A.C.); ansubisakha@gmail.com (A.S.); luigi.palmieri@uniba.it (L.P.); 2 Department of Translational Biomedicine and Neuroscience, University of Bari Aldo Moro, 70124 Bari, Italy; aasiabibi250@gmail.com; 3 Department of Pharmacy, Health and Nutritional Sciences, University of Calabria, 87036 Rende, Italy; vincenza.dolce@unical.it; 4 Department of Cancer Biology and Genetics, College of Medicine, The Ohio State University and Arthur G. James Comprehensive Cancer Center, Columbus, OH 43210, USA

**Keywords:** safe harbor sites, genome, transgenes, gene therapy, CRISPR/CAS9, AAVS1

## Abstract

The stable and safe integration of exogenous DNA into the genome is crucial to both genetic engineering and gene therapy. Traditional transgenesis approaches, such as those using retroviral vectors, result in random genomic integration, posing the risk of insertional mutagenesis and transcriptional dysregulation. Safe harbor sites (SHSs), genomic loci that support reliable transgene expression without compromising endogenous gene function, genomic integrity, or cellular physiology, have been identified and characterized across various model organisms. Well-established SHSs such as AAVS1, ROSA26, and CLYBL are routinely utilized for targeted transgene integration in human cells. Recent advances in genome architecture, gene regulation, and genome editing technologies are driving the discovery of novel SHSs for precise and safe genetic modification. This review aims to provide a comprehensive overview of SHSs and their applications that will guide investigators in the choice of SHS, especially when complementary sites are needed for more than one transgene integration. First, it outlines safety and functional criteria that qualify a genomic site as a safe harbor site. It then discusses the two primary strategies for identifying SHSs: i) traditional lentiviral-based random transgenesis, and ii) modern genome-wide in silico screening followed by CRISPR-based validation. This review also provides an updated catalogue of currently known SHSs in the human genome, detailing their characteristics, uses, and limitations. Additionally, it discusses the diverse applications of SHSs in basic research, gene therapy, CAR T cell-based therapy, and biotechnological production systems. Finally, it concludes by highlighting challenges in identifying universally applicable SHSs and outlines future directions for their refinement and validation across biological systems.

## 1. Introduction

The human genome consists of approximately 3.2 billion base pairs harboring 20,000-25,000 protein-coding genes that make up approximately 1–2% of the entire genome [1,2]. This means that 98–99% of our genome is noncoding and has traditionally been referred to as “junk DNA”, presumed to have only a structural function [3]. However, recent research has revealed that around 80% of the human genome contains functional elements [4]. This includes over 2600 microRNAs (miRNAs), thousands of long non-coding RNAs (lncRNAs), and several classes of transfer RNAs (tRNAs), all playing significant roles in gene regulation [4,5]. Other non-coding functional elements like enhancers, silencers, and promoters orchestrate precise gene expression [6,7]. Repetitive DNA sequences, including short tandem repeats (STRs), contribute to genome variability and can influence gene expression and stability [8]. This uneven distribution of genes and regulatory elements across the genome presents challenges for random transgene insertion, emphasizing the need for precise genome-editing tools to avoid disrupting critical regulatory networks, particularly in genetic engineering and therapeutic contexts [9].

Traditionally, transgene integration has been achieved using retroviral or lentiviral vectors, as well as transposon-based systems such as Sleeping Beauty and PiggyBac, to stably incorporate genetic elements or therapeutic genes into host genomes [10,11]. However, these methods suffer from significant drawbacks. The random integration of transgenes into the host genome poses a significant risk of insertional mutagenesis and disruption of endogenous genes or regulatory elements, potentially leading to oncogenic transformation [12,13,14,15]. Additionally, the integration near regulatory regions can induce transgene inactivation or result in unintended physiological consequences [16]. Furthermore, variations in transgene integration sites among different transgenic cells can also confound phenotypes [17,18]. These drawbacks raise significant concerns about the safety, stability, and reproducibility of random transgene integration methods.

To address these limitations, the concept of Safe Harbor Sites (SHSs), specific loci within the genome that permit stable integration and expression of exogenous genetic material without disrupting native gene function or compromising genomic integrity, has become a cornerstone in the fields of genetic engineering and gene therapy [19,20]. SHSs are typically located in transcriptionally active regions to support the expression of integrated transgenes, but without interfering with the transcription of endogenous genes or increasing the risk of oncogenic transformation [21]. Identification of SHSs has been driven by a combination of computational predictions, empirical studies, and high-throughput screening techniques [22,23]. One of the well-characterized SHSs is the AAVS1 locus on chromosome 19, initially identified as a hotspot for the site-specific integration of adeno-associated virus type 2 and frequently targeted for therapeutic gene integration [24,25,26]. The ROSA26 locus in mice is another well-known and ideal SHS, valued for its permissive and predictable expression profile, together with the absence of a deleterious phenotype when targeted [27,28].

The advent of precise genome-editing technologies, such as CRISPR-Cas9 and TALENs, has greatly enhanced the precise insertion of genetic material into these SHSs, significantly improving the efficiency and reliability of genetic engineering efforts [29,30]. Transgene expression from SHSs ensures controlled and predictable outcomes, mitigating the risks associated with random integration [21,31,32]. Consequently, SHSs provide a robust platform for gene function studies and disease modeling [33], generation of chimeric antigen receptor (CAR) T cells and/or induced pluripotent stem cells (iPSCs) for cell-based therapies [34,35,36], and for the construction of complex genetic circuits with novel functionalities [37]. They also support biopharmaceutical production and the development of genetically modified crops with improved traits while minimizing ecological and biosafety risks [21,35,38].

Despite their advantages, the use of SHSs faces challenges. These include difficulties in identifying universal sites across cell types and tissues due to genomic variability among individuals and species [39]. Risks of off-target effects and unintended alterations during the integration process are possible [40]. Also, the current incomplete annotation, with many genomic regions still poorly characterized and their functional contributions to phenotypic traits still constitute a significant limitation in the use of SHSs [41]. Nonetheless, rapid advances in genome annotation, epigenomics, and single-cell sequencing are enabling the discovery and refinement of SHSs, broadening their biomedical and biotechnological applications [23,42]. Overall, SHSs address limitations of classical transgene integration and open new opportunities in research, therapy, and industry. As our understanding of the human genome deepens and genome editing technologies evolve, SHSs will play a key role in precision medicine and biotechnology. In this review, we provide a comprehensive overview of selection and identification criteria for novel SHSs and the strategies followed to identify them. We discuss in detail the best-characterized SHSs, including those recently discovered, and highlight their applications in fundamental research, gene therapy, and biopharmaceutics production.

## 2. Criteria of SHS Selection

**Figure 1:**
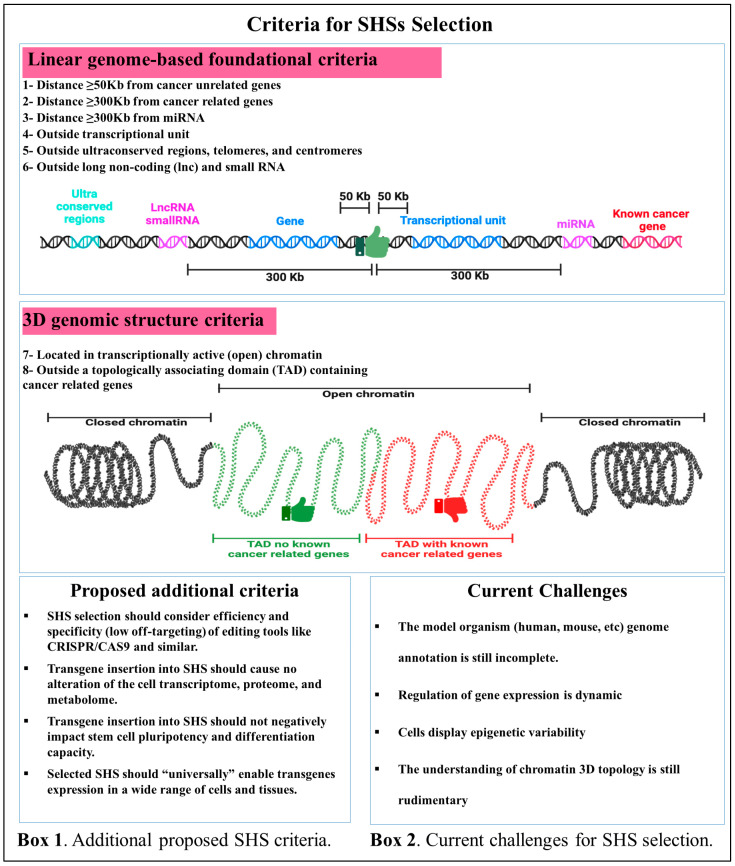
The criteria for SHS identification in the human genome are evolving alongside advances in genome biology. SHS selection criteria can be divided into (i) foundational criteria based on linear genome features, and (ii) criteria based on 3D genome organization and epigenetic hallmarks. Additional criteria are listed to ensure safety, neutrality, and stability of gene expression (BOX 1), and the current challenges in SHS selection are listed as well (BOX 2).

For a genomic site to qualify as a safe harbor, it must fulfill specific criteria to ensure that transgenic elements are stably expressed in the host genome and do not cause alterations such as transcriptional dysregulation of endogenous genes, oncogenes activation and/or tumor-suppressing genes inactivation [43,44]. These criteria have undergone significant revisions in response to advances in our understanding of genome architecture and function. Based on the genomic data and experimental observations obtained from lentiviral vector integration sites, Sadelain and colleagues (2011) proposed five criteria for a candidate site to qualify as a SHS (Figure 1). First, SHSs must be located at a safe distance, typically at least 50 kilobases (kb), from the 5’ end of coding genes in order to prevent the disruption of essential genes that could lead to cellular dysfunctions [45,46]. Second, SHSs must be at least 300 kb away from cancer-associated genes to minimize the risk of oncogene activation [45,46]. Third, they must be at a distance of at least 300 kb from microRNAs [45,46]. Fourth, they must reside outside of the transcriptional units to avoid insertional mutagenesis or transcriptional dysregulation [45,46]. Fifth, SHSs should be outside of ultra-conserved genomic regions, telomeres, and centromeres to avoid interfering with elements critical for gene regulation [45,46].

While these criteria are based on linear genomic features and provide a foundational framework for SHS identification, it is now clear that the genome is organized into three-dimensional (3D) topologically associated domains (TADs) and gene-gene interactions are often confined within these TADs [47,48]. Therefore, the Sadelain group subsequently suggested that SHSs should not be in TADs enriched in oncogenes or regulatory hotspots to provide additional safety against transcriptional activation of proto-oncogenes [21] (Figure 1). However, long-range interactions between genes that are 500 kb away have been reported, underscoring the need for more cautious site selection [15,49]. Moreover, the ever-increasing knowledge about regulatory non-coding RNAs, including long non-coding and small RNAs, now dictates the exclusion of sites that might disrupt these elements [21,50,51]. Finally, in the selection of SHSs, it must be considered that active chromatin characterized by histone modifications, such as H3K4me1 and H3K27ac, favors robust gene expression [52,53]. Therefore, SHSs should be located in the active chromatin regions to allow sustainable transgene expression, possibly across cell types and conditions [21,23] (Figure 1).

The integration of advanced genomic and chromatin epigenetic landscape data into SHS selection criteria introduces both opportunities and challenges. The fine-tuning of SHS definition for specific therapeutic applications enhances safety, but also reduces its generalizability across diverse contexts [45]. As genome-editing technologies like CRISPR-Cas9 evolve, criteria must also continually adapt to account for integration efficiency, off-target effects, and possible chromatin restructuring [54,55]. The efficiency of genomic integration is crucial for clinical applications, where therapeutic success often depends on the expression levels of the therapeutic gene [56]. Off-target mutations could lead to involuntary consequences, including insertional mutagenesis or interruption of essential genes [57,58]. Similarly, targeting SHSs should not significantly alter cell transcriptomics, proteomics, and metabolomics profiles and should maintain iPSCs’ pluripotency, karyotypes and differentiation capacity [19]. Advances in computational modeling, single-cell transcriptomics, and epigenomics hold promise for refining SHS selection criteria [59]. The ultimate goal is to identify universal SHSs enabling robust transgene expression across diverse tissues, cell types, and physiological conditions (Figure 1, BOX1).

Despite these refinements, the criteria for identifying and validating SHSs remain imperfect for several key reasons (Figure 1, BOX2). First, the annotation of the human genome is still incomplete. Initiatives such as GENCODE have remarkably advanced our understanding of the human transcriptome and non-coding elements, yet many functional genomic regions are still poorly characterized [60,61]. Thus, targeting of SHSs selected based on current annotations could inadvertently disrupt unknown regulatory elements. Second, the dynamic and context-dependent nature of gene regulation complicates SHS selection. In fact, targeting sites deemed safe in one cellular context may cause genetic dysregulation in another due to cell-type-specific enhancers, repressors, or chromatin states. Loci deemed non-essential in certain cell types could still have significant biological roles at the systemic level [62,63]. Epigenetic modifications are highly variable and may change over time or in response to environmental cues, leading to alterations in gene expression. This variability may affect the stability and efficacy of transgene expression, posing challenges for long-term predictability [64,65]. Finally, the role of chromatin topology in defining SHSs is not fully understood. In fact, while TAD boundaries and insulator elements are increasingly recognized as critical regulatory structures, the precise mechanism and extent of their influence on gene expression across various cell types are still under investigation [66,67].

## 3. Identification and Validation of SHSs

**Figure 2:**
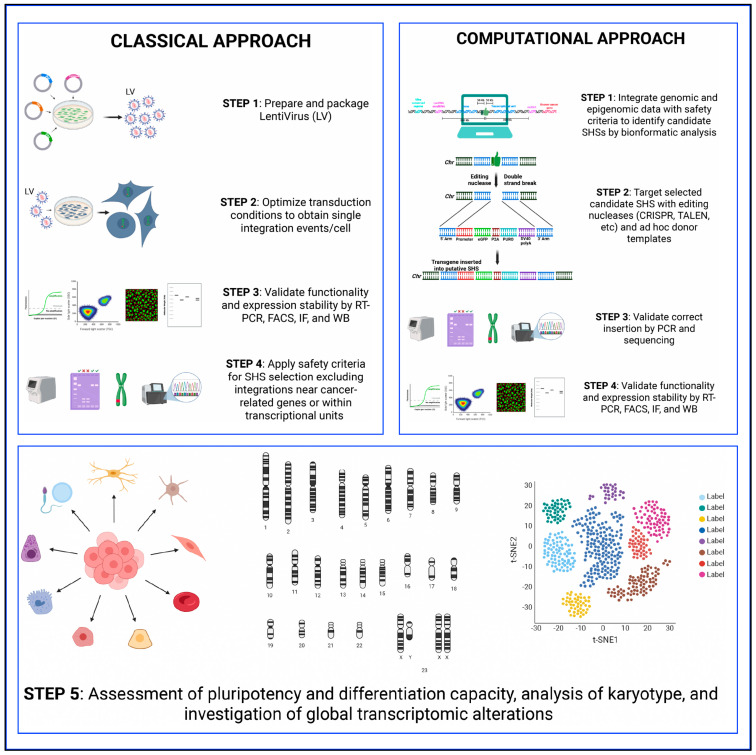
Approaches for the identification of SHSs. Two main strategies are used: (i) a classical approach involving lentiviral vectors to obtain a single integration event per cell, followed by functional validation, integration site mapping, and safety assessment of candidate loci; and (ii) an in silico approach that employs computational tools to scan the genome for candidate sites fulfilling predefined criteria, followed by site-specific nuclease targeting and functional validation.

Identification and characterization of SHSs are fundamental for advancement of genetic engineering, genome editing and gene therapy [68]. Two different but complementary strategies have been developed for novel SHS identification: a classical experimental approach and a computational (i.e., in silico) method [45,51,68,69]. The classical approach primarily relies on the use of retroviral/lentiviral vectors, which integrate semi-randomly into the host genome, preferentially targeting transcriptionally active regions [70]. This inherent bias can be leveraged to identify genomic loci that support stable transgene expression [71,72]. The classical approach involves the optimization of transduction conditions to favor single-copy integrations per cell/genome. The validation of reliable transgene expression includes the use of RT-PCR, flow cytometry, immunofluorescence, and Western blots [45]. This is followed by mapping of integration sites in clones with stable expression, performing inverse and/or ligation-mediated PCR and DNA sequencing [73,74,75]. Finally, candidate loci are evaluated against the SHS safety criteria discussed above to exclude sites occurring in proximity of cancer-related genes, and/or those inside a transcriptional unit that can cause critical alterations in global gene expression as assessed by transcriptomics and proteomics profiling, or in cell karyotype, pluripotency, and differentiation capacity [45,75] (Figure 2). Following this approach, Papapetrou et al. (2011) identified a SHS located in chromosome 1 that met all five foundational criteria and achieved stable β-globin expression in iPSCs derived from thalassemia patients without perturbing neighboring gene expression [45].

The classical approach can also make use of site-specific integrases or homing endonucleases that have numerous pseudo-sites in the human genome. Liu et al. (2009) employed phiC31 integrase to target a GFP reporter cassette into pseudo-attP sites within the human genome, identifying a locus on chromosome 13q32.3 that supported long-term expression in human embryonic stem cells (hESCs) and during neuronal differentiation [76]. Overall, the classical approach enables direct identification and functional validation of candidate SHSs within specific biological contexts and can uncover previously unrecognized loci suitable for safe transgene integration [45,77]. However, it is resource-intensive, requiring careful optimization of transduction conditions and extensive screening to isolate suitable clones that meet the SHS safety criteria and support sustainable gene expression. Additionally, the classical approach is limited by its dependence on the chromatin state of the screened cell type, which may lead to the identification of SHSs that are not active across different cellular contexts [45].

The computational in silico approach employs genome-wide bioinformatics-guided strategies to identify candidate SHSs using genomic and epigenomic datasets [23,51,69]. This strategy typically begins with systematic screening of the genome to locate regions devoid of essential genes, cancer-associated genes, and regulatory elements while favoring integration sites within transcriptionally active chromatin to ensure sustainable transgene expression [23,44,51]. Once candidate loci are identified, the experimental validation starts. Sequence-specific nucleases such as zinc finger nucleases (ZFNs), transcription activator-like effector nucleases (TALENs), or CRISPR/Cas9 are employed to introduce double-strand breaks (DSBs) at the target site. A transgene or reporter gene cassette is then integrated at the break site [51,69,78,79] (Figure 2). The correct site-specific integration is confirmed by PCR using primers flanking the genomic region and the transgene cassette [80,81]. This is followed by functional validation of the integrated genes, e.g., reporter genes, using methods such as RT-PCR, flow cytometry, and Western blot, monitored over an extended period of time to evaluate expression stability and potential silencing [51,69] (Figure 2).

In addition to expression analysis, the safety and biological neutrality of the candidate SHSs are assessed through global transcriptomic profiling. Importantly, the impact on iPSC pluripotency status, karyotypes, differentiation potential, and possible silencing upon differentiation are also considered and assessed [43,51,69]. This comprehensive approach was recently used to identify several new SHSs, such as Rogi1 and Rogi2 [51], Pansio-1, Olonne-18, and Keppel-19 [69], and specific SHSs in blood cell lineages [44]. Compared to the classical experimental approach, the in silico method is efficient, cost-effective, and reduces the number of required experimental iterations by pre-screening genomic regions using computational tools. It can also be applied across various organisms and cell types, provided that high-quality genomic and epigenomic datasets are available. However, computational predictions must be experimentally validated, as they may not fully account for complex biological systems and are limited by the accuracy and resolution of current genome annotations and epigenomic maps.

## 4. Known Genomic Safe Harbors: Features and Limitations

### 4.1. AAVS1

The Adeno-Associated Virus Site 1 (AAVS1), located on chromosome 19q13.4, was originally identified as the preferential genomic integration site for adeno-associated virus type 2 (AAV2) [24,82,83,84]. Integration at AAVS1 is mediated by the AAV Rep protein, which recognizes specific sequence motifs within the site that resemble the viral origin of replication [82,85]. In the absence of Rep, AAV vectors tend to integrate randomly or persist predominantly as episomal DNA [46,86]. AAVS1 resides within intron 1 of the PPP1R12C gene, which encodes a regulatory subunit of myosin protein phosphatase [87]. While PPP1R12C (Protein Phosphatase 1 Regulatory Subunit 12C) is ubiquitously expressed across various human tissues and primary cells, its specific biological function remains incompletely characterized [87]. As SHS, earlier investigations showed that AAVS1 allowed efficient transgene expression in hESCs and iPSCs, and their differentiated progeny without impairing pluripotency and differentiation capacity [88,89]. Subsequent work extended its use to various cell lines, including K562, HeLa, A549, HEK293, U2OS, and mesenchymal stem cells, for applications such as transgene expression and shRNA-mediated gene silencing [90,91,92]. With the advent of CRISPR-Cas9 technology, targeting AAVS1 for transgene expression has become a routine laboratory tool for gene function studies, disease modeling, and therapeutic gene delivery [93,94,95,96,97]. However, emerging evidence indicates that AAVS1 targeting is not without limitations. Silencing and/or expression variability of genes integrated at this locus has been observed in iPSC-derived cardiomyocytes [98,99], endothelial cells [98], myeloid cells [100], hepatocytes [101] and Jurkat T cells [102]. Additionally, transgene integration at AAVS1 perturbed endogenous gene expression, including PPP1R12C [103,104], and neighboring genes TNNI3 (Troponin I3) and PPP6R1 (Protein Phosphatase 6 Regulatory Subunit 1) [23]. Furthermore, given that AAVS1 lies in a gene-dense genomic region, the potential for unintended dysregulation of additional neighboring genes remains underexplored and requires further investigation (Table 1).

### 4.2. hROSA26

The human homolog of murine Rosa26 locus (hROSA26) was identified by searching the human genome using the mouse ROSA26 transcript as a template [27,105]. The search resulted in a region with high sequence similarity (85%) on human chromosome 3 (3p25.3) in the intron 2 of THUMPD3 (THUMP domain 3 tRNA Guanosine Methyltransferase) gene, and hROSA26 mRNA was detected in various hESCs and tissues [27,106]. *THUMPD3*, in complex with *TRMT112* (tRNA methyltransferase activator subunit 11-2), functions as an N^2^-methylguanosine (m^2^G) methyltransferase that modifies specific residues in a broad range of cytoplasmic tRNAs, thereby playing a significant role in translation regulation and cellular growth [107]. Targeting the hROSA26 locus with red fluorescent protein (RFP) reporter cassette permitted stable expression in hESCs [27]. Subsequent recombinase-mediated cassette exchange (RMCE) successfully replaced RFP with a puromycin resistance gene, demonstrating the locus’s amenability to efficient and versatile genome engineering [27]. Importantly, hESCs targeted at the hROSA26 locus retained typical stem cell morphology, alkaline phosphatase activity, normal karyotype, and the capacity to differentiate into neurons, chondrocytes, smooth muscle cells, and hepatocytes [27,108]. They also preserved the capacity to differentiate into the three germ layers, ectoderm, endoderm and mesoderm, and trophoblast lineage [27]. Similarly, hiPSCs targeted at the hROSA26 locus were differentiated into microglia-like cells (MGLs), which exhibited canonical morphological, transcriptional, and functional properties of native microglia in monoculture, co-culture, and 3D organoid models [109]. The locus has also been utilized for stable transgene expression in various human cell lines, including hTERT-RPE1 (hTERT-immortalized Retinal Pigment Epithelial 1) [110], and HEK293 (Human Embryonic Kidney 293) cells [111,112]. Collectively, these findings establish hROSA26 as a locus suitable for stable and consistent transgene expression across multiple human cell types, making it a valuable platform for genetic engineering and biomedical research. Nevertheless, this locus, similar to AAVS1, is situated in a gene-rich region, raising concerns about potential disruption of nearby genes upon transgene integration [45]. Furthermore, since the locus lies within intron 2 of *THUMPD3*, a gene whose function is not yet fully understood, the long-term consequences of its perturbation remain unclear (Table 1). A comprehensive transcriptomic analysis following hROSA26 targeting is currently lacking, and further investigation to assess the broader impact of genetic manipulation at this locus is needed.

### 4.3. H11 Locus

The human Hippo 11 (H11) locus is located on chromosome 22q12.2 within a transcriptionally active intergenic region flanked by the *DRG1* (Developmentally Regulated GTP Binding Protein 1) and *EIF4ENIF1* (Eukaryotic translation Initiation Factor 4E Nuclear Import Factor 1) genes. It was identified through comparative genomic analysis using the murine orthologs of *DRG1* and *EIF4ENIF1*, which revealed a conserved gene arrangement and intergenic distance between the two genes in both species, despite only ~45% sequence identity and absence of highly conserved elements [113]. This locus is situated approximately 4500 and 683 bp downstream of *DRG1* and *EIF4ENIF1*, respectively, as both genes are transcribed from the opposite strands [113]. The H11 locus fulfills certain safety criteria, including absence of known oncogenes and microRNAs within a 300 kb window while residing in a region with high transcriptional activity [113]. Targeting this locus supported stable expression of various transgenes in hiPSCs, including transcription factors (*LMX1A*, *FOXA2*, and *OTX2*), the auxin-inducible degron component *TIR1*, and coagulation factor VIII, even after extensive passaging and differentiation, with no evidence of transgene silencing [113,114,115] (Table 1). Additionally, the H11 locus allowed recombinase-mediated cassette exchange (RMCE) using *phiC31* and *Bxb1* integrases in both healthy and patient-derived hiPSCs [113,115]. Importantly, targeted hiPSC cells maintained typical pluripotent morphology, expressed canonical pluripotency markers, preserved normal karyotypes, and readily differentiated into derivatives of all three germ layers (endoderm, mesoderm, and ectoderm), confirming their intact developmental potential [113,114]. In summary, the H11 locus represents a promising intergenic SHS for stable transgene integration, supporting high and consistent expression without impairing stem cell pluripotency or differentiation capacity. However, the effect of targeting this locus on global transcriptomics profiling and the expression of neighboring genes is not known and requires further investigation.

### 4.4. CCR5 Locus

CCR5 (C-C motif chemokine receptor 5) gene, located on chromosome 3p21.31, encodes for a receptor involved in the immune response [116]. CCR5 is best known for its role as a co-receptor facilitating Human Immunodeficiency Virus (HIV) entry into host cells [116]. The interest in CCR5 as a potential SHS site for transgene integration stemmed from studies of individuals carrying a 32-base pair deletion (CCR5-∆32). This naturally occurring mutation renders CCR5 non-functional and confers resistance to HIV infection without causing significant adverse effects [117,118,119]. These findings suggest that CCR5 can be disrupted without detrimental consequences, making it an attractive candidate for targeted transgene integration. Experimental disruption of the CCR5 gene in hiPSCs did not alter the expression of pluripotency markers or impair differentiation capacity into the three germ layers and CD34^+^CD43^+^ hematopoietic progenitors, and maintained a normal karyotype [120,121]. The ZFN-mediated GFP gene integration into the CCR5 locus in hESCs resulted in stable expression without affecting their pluripotency or differentiation capacity into neural progenitors [122]. Furthermore, chimeric antigen receptor (CAR) genes integrated into the CCR5 locus in T cells enabled stable transgene expression, conferred them with resistance to HIV infection, while allowing these modified T cells to target HIV-infected cells [123]. Despite its potential as a SHS, certain challenges have been encountered when using the CCR5 locus for transgene expression, including inherently low expression likely due to heterochromatin markers and/or gradual decline in transgene expression from CCR5 [35,124,125]. Targeting the CCR5 locus has also been associated with upregulation of neighboring genes, including CCR1 and CCR3 [125]. Importantly, disruption of CCR5 increases the susceptibility to West Nile virus infection that can cause fatal encephalitis [126,127] (Table 1).

### 4.5. Citrate Lyase Beta-Like (CLYBL)

CLYBL locus, mapped to intron 2 of the Citrate Lyase Beta-Like (CLYBL) gene on the long arm of chromosome 13 (13q32.1), was initially identified as a potential SHS site through phiC31 integrase-mediated targeting of a GFP reporter into a pseudo-*attP* site in this region [128]. The CLYBL gene encodes citramalyl-CoA lyase, a ubiquitously expressed mitochondrial enzyme with malate/β-methylmalate synthase activity, involved in the C5-dicarboxylate metabolic pathway, including itaconate metabolism. Loss of CLYBL gene function has been associated with vitamin B12 deficiency [129,130]. Transgenes integrated into the CLYBL locus in hiPSCs and neuronal stem cells (NSCs) yielded stable and superior expression to that achieved from the AAVS1 site [104] (Table 1). Importantly, the transgene integration at the CLYBL locus caused less disruption to local gene expression compared to other loci. For instance, while targeted transgene integration into the AAVS1 locus showed significant downregulation of the PPP1R12C gene (>1000-fold) and upregulation of other neighboring genes, targeting the CLYBL locus resulted in only a ~50-fold decrease in CLYBL expression with minimal impact on neighboring gene activity [104]. Numerous studies have shown that targeted gene integration into CLYBL site of hESCs and iPSCs not only supports a strong transgene expression but also preserves the parental karyotype, typical stem cell morphology, expression of pluripotency markers (Oct4, SSEA4, Tra1-60, and Tra1-8), while maintaining the capability to differentiate into various lineages (e.g., neurons, microglia-like cells, astrocytes) and to form all three germ layers [104,128,131,132,133,134,135]. The functional utility of this locus has also been demonstrated in HeLa and HEK293 cells [135]. This locus has also successfully been targeted using exchangeable DNA payloads, enabling insertion of various transgenes for diverse research applications [136]. These findings underscore the CLYBL locus’s potential as a valuable site for safe and efficient transgene integration, particularly in the context of stem cell research and therapeutic applications. Its favorable genomic context and minimal impact on surrounding gene expression enhance its appeal as a safe locus. However, the long-term stability of transgene expression and the impact on the global transcriptomics profile upon CLYBL targeting remain to be assessed.

### 4.6. SHS231

The homing endonuclease mCre1 is a class of enzymes that recognize and cleave a specific 20 bp DNA sequence to catalyze the lateral transfer of parasitic DNA elements [137]. Comprehensive studies have demonstrated that mCreI can still cleave DNA sites with a single base substitution (19/20 bp identity, or 95%) at approximately 90% efficiency [137,138,139]. Pellenz and colleagues searched the human genome for potential mCreI pseudo-sites, including those with one base-pair substitution (19/20 bp) [80,140]. This analysis identified 35 sites across 16 chromosomes, including both arms of the X chromosome, and in vitro studies confirmed that mCreI could cleave these sequences [80]. These candidate sites were evaluated using eight SHS criteria to assess safety, functionality, and structural accessibility and were compared to the established SHSs, i.e., AAVS1, CCR5 and hROSA26. Notably, ten of the mCreI target sites met 6–7 of the eight core SHS criteria, whereas AAVS1, CCR5, and hROSA26 met only 5, 5, and 3 criteria, respectively [80]. One promising site, designated as SHS231, located on the long arm of chromosome 4, met 7 out of the 8 SHS criteria. This site was targeted using CRISPR-Cas9 for both homology-dependent and non-homology-dependent transgene integration in various rhabdomyosarcoma (RMS) cell lines (Rh5, Rh30, and SMS-CTR) as well as in HEK293T cells [80]. Targeting the SHS231 site enabled GFP expression for more than 45 days in Rh5 and SMS-CTR cells, indicating robust integration and stable transgene expression [80]. Additionally, when targeted with a Cas9 expression cassette, the SHS231 site supported stable Cas9 expression, enabling functional genome editing, for instance, (i) Cas9 mediated a 17 kb deletion in the PAX3/FOXO1 fusion oncogene in Rh30 cells, and (ii) Cas9-VPR integrated at SHS231 activated the MYF5 promoter in RMS cells, inducing robust expression [80]. These findings suggest that SHS231 is a novel and promising SHS that can be targeted in various human cell lines for application in gene therapy, functional genomics, and disease modeling [80] (Table 1). More recently, Vlassis et al. demonstrated that four additional sites identified in the Pellenz study, namely SHS257 (chr7), SHS325 (chr8), SHS313 (chrX), and SHS301 (chr7), also enabled efficient and stable expression in hiPSCs, primary T and natural killer (NK) cells and in Jurkat cells [141]. These sites supported sustained transgene expression throughout the differentiation of iPSC toward CD34^+^ hematopoietic stem and progenitor cells, lymphoid progenitor cells, and NK cells [141]. However, additional studies are required to assess global transcriptomic alterations and the long-term stability of transgene expression upon targeting these novel SHSs.

**Table 1:** A summary of SHSs in human genome and their features.

SHS Locus	Chrom- osome	Intergenic/ Intragenic	Cell Models	Transgene Expression	Effect on Stem Cells Characteristics	Effect on Transcriptomics	Reported Silencing	Gaps and Limitations	Refs
AAVS1	19q13.42	Intragenic PPP1R12C	Many cellular models, including hESCs	Robust expression	No effect on hESCs’ pluripotency and differentiation capacity	Unknown	Yes	Located within PPP1R12C transcriptional unit and in a gene-dense region	[87]
hROSA26	3p25.3	Intragenic THUMPD3	Many cell models, including hESCs	Robust expression	No effect on hESCs’ pluripotency and differentiation capacity	Unknown	No	Located within THUMPD3 gene transcriptional unit and in a gene-dense region	[27]
H11	22q12.2	Intergenic DRG1-H11-EIF4ENIF1	Mainly in hiPSCs	Robust expression	No effect on hESCs’ pluripotency and differentiation capacity	Unknown	No	Located between two genes whose dysregulation upon targeting is not known	[113,114,115]
CCR5	3p21.31	Intragenic CCR5 gene	hESCs, T cells and HEK293T cells	Low expression	No effect on hESCs’ pluripotency and differentiation capacity	Unknown	Yes	Upregulated the flanking genes CCR1 and CCR3, and increased the susceptibility to West Nile virus infection	[122]
CLYBL	13q32.1	Intragenic CLYBL gene	iPSCs, hESCs, NSC and in Hela and HEK293 cells	Robust expression	No effect on hESCs’ pluripotency and differentiation capacity	Unknown	No	Located within coding gene, and the long-term transgene expression is not known	[104,132]
SHS231	4q13.1	Intergenic	Rh5, Rh30, SMS-CTR, and HEK293T	Robust expression	Unknown	Unknown	No	Transgene expression in other cell models, like iPSCs, is not assessed	[139]
Rogi1	1q31.3	Intergenic	HEK293T, Jurket, and primary T cells and iPSCs	Robust expression	Unknown	Minimal effect	Yes	The effect on stem cells’ pluripotency, karyotype, and differentiation capacity is not assessed	[51]
Rogi2	3p24.3								
Pansio-1	1p13.2	Intergenic	hESCs and iPSCs	Stable expression	No effect on hESCs’ pluripotency and differentiation capacity	Minimal effect	No	The long-term stability and transgene expression in other cell models are not assessed	[69]
Olonne-18	18q21.31	Intergenic							
Keppel-19	19p13.3	Intergenic							
BLD_SHS10	3p22.2	Intronic GOLGA4	HUDEP2 erythroid progenitors	Stable expression	Unknown	Unknown	No	These sites may be targeted in HSCs to assess the impact on HSC physiology and differentiation capacity into diverse blood cell lineages.	[23]
BLD_SHS14	6q25.3	Intronic ARID1B							
BLD_SHS15	8q24.12	Intronic/ TAF2							
eSHS6	7q36.1	Pseudogene ZNF767P	T cells	Stable expression	Unknown	Unknown	No	Functionality of this locus in other blood cells and iPSCs warrants further assessment.	[44]

### 4.7. Rogi1 and Rogi2

Aznauryan and colleagues employed a computational approach to identify novel SHSs suitable for safe transgene integration and stable expression [51]. Their strategy excluded sites located in gene-dense regions, or within transcriptional units, and those near oncogenes, microRNAs, long non-coding RNAs, tRNAs, or transcriptional enhancers. Additionally, candidate sites were also required to be at least 300 kb away from telomeres and centromeres [51]. This stringent filtering process yielded over 2000 potential SHSs distributed across the human genome that satisfied the defined safety criteria. Among these, two sites named as Rogi1 and Rogi2 (Region for optimal gene insertion 1 and 2) localized on chromosomes 1 and 3, respectively, were experimentally validated as SHSs. Both sites enabled robust and sustained expression of the reporter gene mRuby for over 90 days in HEK293T and Jurkat cell lines, outperforming traditional SHSs such as AAVS1 and CCR5 [51] (Table 1). Similarly, in human primary T cells, integration into Rogi1 and Rogi2 supported stable mRuby expression for over 20 days. In human dermal fibroblasts, expression of the therapeutic gene *LAMB3* from both sites remained sustained for at least 25 days [51]. Transcriptomic analysis of HEK293T and Jurkat cells harboring mRuby at the Rogi2 locus revealed a preserved global transcriptional profile [51]. Similarly, integration of mRuby into Rogi1 in primary human T cells resulted in minimal transcriptional alteration, as evidenced by single-cell RNA-seq analysis. Importantly, no cancer-related pathways were upregulated; in fact, the JUN oncogene, which encodes the c-Jun transcription factor, was downregulated [51]. Of note, reporter gene expression displayed clonal heterogeneity in HEK293T and Jurkat cell lines, which is likely attributable to the inherent genomic instability of immortalized cancer cells [51]. A recent study targeted the transcription factor FOXN1 gene into the Rogi1 site in iPSCs and subsequently differentiated them into thymic epithelial cells upon FOXN1 induction [142]. However, this work reported the silencing of the tetracycline repressor and leakage of FOXN1 expression, which were mitigated by introducing an A2-ubiquitous chromatin opening element (A2UCOE) and an SV40 poly(A) termination sequence upstream of the tetracycline-inducible promoter [142]. It remains unclear whether this silencing and leakage are attributable to the genomic locus used or are intrinsic properties of the cassette. Furthermore, the effects on pluripotency, karyotype, and differentiation potential are not widely assessed yet and warrant further investigation.

### 4.8. Pansio-1, Olonne-18, and Keppel-19

Autio and colleagues employed a computational strategy to identify new SHSs in the human genome that fulfill the established SHS selection criteria [69]. The analysis resulted in the identification of 25 putative SHS loci. Three of them-Pansio-1, Olônne-18, and Keppel-19-were experimentally validated in hESCs (Table 1) [69]. Targeted transgene integration at these loci had a minimal effect on the native transcriptomic profiling and caused no karyotype or pluripotency abnormalities [69]. Furthermore, targeting these sites did not impair the iPSCs’ capacity to differentiate into neuronal, liver, cardiac, and pancreatic β-cells or to give rise to endoderm, mesoderm, and ectoderm tissues [69]. Importantly, the expression of MAGI3, TXNL1, and ZNRF4 genes closest to Pansio-1, Olônne-18 and Keppel-19, respectively, was not significantly altered following transgene integration. Additionally, the transgene expression from these loci remained stable for at least 15 passages and was maintained following differentiation into neuronal, hepatic, and cardiac lineages [69]. Notably, these loci enabled sustainable transgene expression both from constitutive and inducible (Tet-On) promoters integrated into these sites and supported Bxb1 integrase-mediated cassette exchange [69,143]. While these findings highlight the potential of Pansio-1, Olônne-18, and Keppel-19 as valuable SHS sites, further research is needed to assess long-term transgene stability, validate these loci across additional cell types, and confirm their performance in in vivo models.

### 4.9. Cell/Tissue Specific SHSs

Traditional approaches for identifying SHSs have primarily focused on static genomic features, often overlooking tissue-specific genome architecture, transcriptional activity, and regulatory elements [51,69,144]. Incorporating 3D chromatin organization in a tissue-specific manner into SHS identification methods can help select a more refined, cell-type-specific SHS that supports stable transgene expression without disrupting essential genes or interfering with native transcriptional programs [23]. Shrestha et al. analyzed polymorphic mobile element insertion sites, epigenomic signatures, and 3D chromatin structure using data from the 1000 Genomes Project and the Genotype-Tissue Expression project. Their aim was to identify loci where mobile elements had inserted without detrimental effects, suggesting potential tolerance for additional genetic material [23]. This integrative approach led to the identification of 19 unique SHSs in blood cells (designated as BLD_SHS1-BLD_SHS19) and 5 in brain cells (designated as BRN_SHS1-BRN_SHS5). Most of these sites were located in the intronic regions, and only one (BLD_SHS_7) was found in an intergenic region. All SHSs were mapped into active chromatin regions, and 13 were located outside of TADs with high gene-density or in TADs harboring cancer-related genes, particularly in the lymphoblastoid GM12878 cell line. Among these, BLD_SHS10 (chr3:37361602–37361603) resides in the intron of the GOLGA4 gene, which is the sole gene within its relative TAD [23]. Three candidate sites, BLD_SHS10, BLD_SHS14, and BLD_SHS15, were experimentally validated in HUDEP2 erythroid progenitors, demonstrating that transgenes integrated at these sites exhibited stable expression over several weeks and after differentiation into mature erythrocytes, without significantly disrupting the expression of nearby genes [23] (Table 1). These findings indicate that the newly identified loci may serve as tissue-specific SHSs for therapeutic gene integration, potentially enhancing the safety and precision of gene and cell-based therapies [23].

Odak et al. identified a novel extragenic SHS site that can be used for precise therapeutic T-cell engineering. They employed a computational and experimental framework to identify and validate SHSs in human peripheral T cells that satisfy key safety and efficiency criteria [44]. Specifically, the assay for transposase-accessible chromatin coupled to high-throughput sequencing (ATAC-seq) was used to identify regions of open chromatin in T cells, revealing 379 candidate sites characterized by strong ATAC-seq peaks and that fulfilled SHSs safety criteria [44]. Among them, an extragenic site designated as eSHS6 localized to chromosome 7, within the pseudogene ZNF767P, showed high cleavage efficiency (>80% when targeted with CRISPR/Cas9) and chromatin accessibility [44] (Table 1). Human peripheral blood T cells were targeted with CRISPR/Cas9 and a donor vector to integrate a CD19-CAR construct into the eSHS6 site and then evaluated in vitro and in vivo for CAR expression and T-cell function [44]. The resulting eSHS6-CAR T cells maintained durable CAR expression over multiple weeks of antigen stimulation and provided long-term leukemia control in vivo [44]. eSHS6-CAR T cells maintained robust anti-tumor activity even after multiple leukemia rechallenges and demonstrated proliferation, persistence, and tumor clearance similar to T-Cell Receptor Alpha Constant (TRAC)-CAR T cells [44]. Noticeably, eSHS6-CAR T cells, unlike TRAC-CAR T cells, retained their native T-cell receptor, which could be beneficial for additional antigen recognition and expansion [44]. Thus, this study represents the first systematic identification and functional validation of extragenic SHSs in human T cells, highlighting eSHS6 as a promising alternative to TRAC for CAR integration for safer and more versatile T-cell-based immunotherapies [44].

## 5. Safe Harbor Sites Applications

### 5.1. SHSs’ Uses in Basic Research

Targeted integration of genetic material into well-characterized SHSs offers a powerful strategy for achieving controlled and predictable gene expression for functional studies, without disrupting endogenous gene function [21,145]. Unlike traditional lentiviral methods, which are prone to random insertion and position-dependent variation, SHS-targeted integration provides consistent transgene expression, thereby enhancing the reliability and reproducibility of experimental outcomes [20]. This approach has enabled a wide range of innovative applications. For instance, the SHS for transgene expression in iPSCs is used for the generation of 3D organoid models to accurately study complex biological processes [145]. Indeed, human iPSCs engineered to express channelrhodopsin-2 (ChR2) from the AAVS1 locus and differentiated into forebrain organoids exhibited a robust neural activation upon light stimulation [87]. When these organoids were connected with spinal cord and skeletal muscle organoids, they triggered consistent muscle contractions, offering a robust model to study brain function and brain-periphery neural circuits [87]. Similarly, Zhang et al. targeted hiPSCs at the AAVS1 locus with a voltage-sensitive fluorescent protein, enabling real-time action potential recordings in differentiated ventricular, atrial, and nodal cardiomyocytes in both 2D and 3D cultures. This non-invasive approach provides a scalable alternative to traditional patch-clamp techniques for electrophysiological studies [146]. Integration into SHSs can also be used for the creation of reliable disease models. Dost et al. engineered hiPSCs to express the oncogenic *KRAS^G12D^* from the AAVS1 locus, generating an early-stage lung adenocarcinoma organoid. These organoids showed a loss of differentiation markers in alveolar type 2 cells upon *KRAS* activation, mimicking both mouse models and human tumor samples [147]. SHSs can also be used to integrate lineage-specific transcription factors in hiPSCs, which can be induced to facilitate direct differentiation into various cellular lineages such as neurons and myoblasts, avoiding the use of cost- and time-consuming protocols. In this regard, human iPSCs targeted at the AAVS1 and CLYBL loci with an open reading frame encoding the transcription factor PAX7 were readily differentiated into expandable myogenic progenitors capable of robust engraftment upon induction of PAX7 expression [148,149]. Also, hiPSCs targeted with the neuronal transcription factor neurogenin 2 at the AAVS1 locus differentiated into mature neurons upon the induction of neurogenin 2 expression [150,151,152,153]. SHS transgene integration also enables the generation of cellular models that can be used for functional genomics, gene-gene interaction, gene dependency mapping, and high-throughput drug screening studies [108,154]. Landin et al. developed hiPSCs and mouse models expressing CRISPR/Cas9 from the AAVS1 and murine ROSA26 loci, respectively [155]. When these mice were transduced with AAV vectors carrying guide RNAs and repair templates specific to *KRAS*, *TP53*, or *STK11*, they developed lung adenocarcinomas that faithfully recapitulated human tumor pathology and drug response, highlighting the model’s translational relevance [155]. In summary, those pioneering studies point to SHSs as invaluable tools for basic research.

### 5.2. SHSs for CAR T Cell-Based Therapy

CAR T-cell therapy is an immunotherapeutic approach in which a patient’s T cells are genetically engineered to express CARs that selectively recognize and eliminate cancer cells [156]. This personalized treatment harnesses the body’s own immune system to target especially hematologic malignancies such as acute lymphoblastic leukemia (ALL) and multiple myeloma [157]. Ongoing efforts are also exploring its potential in treating solid tumors and infectious diseases [158]. Conventional CAR T-cell production relies on random transgene integration, often resulting in variable and suboptimal expression [159,160]. Targeted insertion of CAR cassettes into SHSs offers a powerful alternative, enabling consistent expression and improved safety profiles [161,162,163,164,165]. Indeed, CAR T-cells engineered to express an anti-CD105 nanobody from the AAVS1 site displayed hallmark features of activated cytotoxic T-cells when co-cultured in vitro with CD105-positive cancer cells: they proliferated, secreted pro-inflammatory cytokines, and efficiently eliminated target cells in vitro. In vivo, these cells markedly inhibited the growth of CD105-positive tumors and prolonged survival in tumor-bearing NOD/SCID mice [166]. Similarly, the CD19-CAR construct targeted to the eSHS6 site mentioned above showed sustainable CAR expression with or without antigen stimulation, effectively lysed CD19^+^ NALM6 cells, and provided long-term tumor control in an NSG (NOD/SCID/IL-2Rγ null) model of B-cell ALL [44]. In a different study, anti-CD19 CAR cassettes containing 4-1BB and CD3ζ signaling domains were integrated into the AAVS1 site or PD-1 gene locus of T cells. These engineered T cells eradicated tumor cells in xenograft mouse models [162]. Interestingly, in a clinical trial of eight patients with relapsed/refractory aggressive B-cell non-Hodgkin lymphoma, engineered cells achieved a strong (87.5%) remission rate without severe adverse events even at a low infusion dose [162]. 

SHSs can also be exploited to engineer immune cells other than T-cells to exert a specific function. For instance, hiPSC-derived neutrophils engineered to express a CAR targeting the prostate-specific membrane antigen from the AAVS1 locus showed a potent cytotoxic effect against the prostate LNCaP cell line in vitro [161]. Similarly, hiPSCs modified to express CARs from the AAVS1 or H11 site were differentiated into CAR macrophages that efficiently phagocytosed tumor cells in vitro and exhibited potent antitumor effects in mouse models of ovarian cancer and neuroblastoma [167,168]. SHS targeting can also be explored for engineering CAR cells to combat infectious diseases. Single-chain variable fragments (scFvs) derived from broadly neutralizing anti-HIV antibodies, integrated into the T cell CCR5 locus, conferred potent lytic activity against HIV-infected cells both in vitro and in a PBMC-humanized mouse model of HIV infection [123]. Similarly, integration of an anti-CD19 CAR into the CCR5 site generated T cells that are resistant to HIV infection while maintaining robust antitumor activity against HIV-associated B-cell malignancies in vitro and in vivo [169]. Altogether, these studies suggest that precise CAR integration into SHSs enhances expression stability, functional potency, and biosafety across diverse immune cell types. This strategy represents substantial advances over random integration, offering greater predictability and durability for CAR-based therapies against both cancer and infectious diseases. However, significant challenges remain in the efficient isolation of primary blood cells, particularly T cells, as well as in their ex vivo expansion and genetic modification, which can affect cell viability, editing efficiency, and long-term therapeutic function.

### 5.3. Use for Targeted Gene Therapy

Gene therapy using SHSs has emerged as a transformative strategy for treating genetic disorders, cancers, and chronic diseases with significant advantages over traditional approaches using lentiviral vectors [170,171,172,173,174,175,176]. Several proof-of-concept studies highlight the therapeutic potential of SHS targeting. In primary hyperoxaluria type 1 (PH1), insertion of the alanine–glyoxylate aminotransferase (AGXT) gene into the AAVS1 site of PH1 patient-derived iPSCs enabled stable AGXT expression both in iPSCs and differentiated hepatocyte-like cells, suggesting their potential use in autologous cell-based gene therapy for PH1 treatment [174]. In mucopolysaccharidosis type I (MPSI), integration of the iduronidase (IDUA) gene into the CCR5 locus of human CD34^+^ hematopoietic stem cells (HSCs) led to high levels of the IDUA enzyme secretion, maintained capacity to differentiate into multiple blood cell lineages, and improved both biochemical and phenotypic abnormalities in an immunocompromised MPSI mouse model [177]. In X-linked chronic granulomatous disease, the insertion of the CYBB gene (encoding the gp91^phox^ subunit of NADPH oxidase) into the AAVS1 site of patient-derived CD34^+^ HSCs restored gp91^phox^ expression and NADPH oxidase activity in ex vivo–derived neutrophils [178]. When these gene-corrected HSCs were transplanted into NSG mice, 4–11% of bone marrow-derived human cells maintained gp91^phox^ expression [178]. Similarly, the integration of coagulation factor VIII (FVIII) into the AAVS1 locus of hemophilia A (HA) patient-derived iPSCs supported stable FVIII expression in their endothelial derivatives. Transplantation with these engineered endothelial cells into an immunocompetent HA mouse model resulted in a 2.12-fold increase in plasma FVIII activity and improved survival following haemorrhagic injury [179]. Similarly, hiPSCs derived from a hemophilia B patient with the targeted insertion of the coagulation factor IX (F-IX) into the AAVS1 locus readily differentiated into hepatocytes capable of stable F-IX secretion and short-term functional activity after engraftment into NOD/SCID mice [180]. Collectively, these studies underscore the potential of SHSs as valuable tools for targeted gene therapy. Nonetheless, the emergence of more precise gene editing technologies like base and prime editors capable of precisely correcting pathogenic mutations will likely limit the use of SHSs for certain gene therapy applications.

### 5.4. Biotechnology Applications

Pharmaceutical proteins, including hormones, vaccines, enzymes and antibodies, are typically produced using genetically engineered microbial cells or mammalian cell lines like Chinese hamster ovary (CHO) cells and HEK293 cells targeted with transgene cassettes using lentiviral and retroviral vectors [181,182,183,184,185]. SHSs represent now the tool of choice for therapeutic protein expression as they allow robust and stable expression over extended culture periods [124]. In this regard, Deng et al. utilized CRISPR/Cas9 to integrate human serum albumin (HSA) and interferon-β-HSA fusion (IFNβ-HSA) into hROSA26, AAVS1, and CCR5 loci of HEK293T and assessed their stability in high-density suspension culture [35]. The expression from the hROSA26 locus proved the most stable, maintaining > 98% of transgene expression after 60 passages, while that from the CCR5 site showed reduced expression, likely due to transcriptional silencing [35]. The study also demonstrated that the highest yield of HSA and IFNβ-HSA was obtained from hROSA26-engineered cells [35]. Comparable yield was also achieved targeting the AAVS1 locus, whereas insertion at the CCR5 yielded a markedly low amount. Although the obtained yields remain insufficient for industrial use, targeting multiple loci within the same engineered cell line combined with optimized culture conditions may enhance productivity [35]. In this regard, Yang et al. integrated the CTLA4-Ig fusion, made of the cytotoxic T-lymphocyte-associated protein 4 and the IgG1 Fc fragment used for immunosuppression, into the AAVS1 locus of HEK293T cells, achieving stable protein production [186]. Notably, HEK293 cells are advantageous for biopharmaceutical protein manufacturing because they are of human origin: they are able to produce proteins with proper folding and post-translational modifications [187,188]. Also, HEK293 cells are easy to manipulate, they readily domesticate in suspension, and, unlike CHO cells, do not usually produce clipped protein products [187,188]. In summary, SHSs are the tool of choice for biopharmaceutical protein production due to their transgene expression homogeneity and stability. However, both identifying the optimal loci and refining culture conditions remain a critical challenge for achieving industrial-scale protein synthesis.

### 5.5. Other Applications

SHSs are increasingly leveraged for a wide range of innovative and interdisciplinary applications in both clinical and non-clinical settings. SHSs provide an ideal genomic environment for the stable integration of synthetic gene circuits. This is particularly valuable in engineering mammalian cells to perform complex tasks such as environmental sensing and synthesis of pharmaceuticals [189]. Targeting synthetic elements into SHSs ensures uniform expression across cell populations, reducing variability and positional effects that can compromise circuit function [189]. SHSs also offer strategic advantages for generating transgenic plants and animals with enhanced traits, disease resistance, or improved nutritional profiles, while minimizing the risks of insertional mutagenesis and transgene silencing [190,191]. In addition, they can serve as secure integration sites for biosensors and reporter genes designed to detect metabolic cues, track biological processes, or monitor environmental toxins and pathogens [192,193]. Stable sensor expression from SHSs ensures consistent functionality across cell generations, a feature essential for reproducibility and regulatory compliance [193,194]. Altogether, these emerging applications place SHSs as versatile platforms for safe and stable transgene expression. As more context-specific SHSs are identified, their utility in next-generation genomic engineering will continue to expand substantially.

## 6. Conclusions, Challenges, and Future Perspective

SHSs represent a pivotal advancement in the field of transgenesis, genetic engineering, and gene therapy. By providing reliable genomic locations for transgene integration with minimal disruption to native gene function or genomic integrity, SHSs have become invaluable tools for diverse applications from studying gene function and modeling human diseases to supporting drug discovery, producing biopharmaceuticals, and building genetic circuits for complex bioprocesses.

Despite their promises, the implementation of SHSs for a wider range of applications is hindered by several challenges. First, identifying and validating SHSs requires extensive genomic and epigenetic profiling to ensure they do not interfere with essential regulatory elements. This is further complicated by our incomplete understanding of the human genome and epigenome, compounded by the fact that epigenetic landscapes vary among individuals and can change in response to environmental factors. Second, epigenetic-mediated transgene silencing remains a major challenge in transgenesis and genetic engineering [16]. Given our limited understanding of human epigenetic regulation, it is difficult to predict or prevent silencing events of SHS-integrated transgenes, which have been reported in several cellular models, including iPSCs-derived cardiomyocytes, hepatocytes, and myeloid cells [100,101,195]. Third, the transgene targeting technologies, such as CRISPR/Cas9, can cause off-target effects with unintended consequences, also due to DNA double-strand breaks required for integration. Fourth, targeted gene integration into SHSs is inherently inefficient and is often outcompeted by faulty DNA repair, leading to insertions or deletions. Fifth, methods for delivering genome editing components together with donor templates remain inefficient, particularly for large inserts, and can induce cytotoxicity. Finally, clinical use of SHSs raises important ethical questions about consent, privacy, and possible social impacts. Especially, germline editing raises concerns over “designer babies” and the broader ethical consequences of altering the human germline.

Future studies should focus systematically on addressing these biological, technical, and ethical challenges while leveraging the constant advances in genome engineering. Integrating multi-omics data, including transcriptomic, epigenomic, and proteomic profiles, will improve the precision and robustness of SHS identification, ensuring the selection of loci that remain consistently active across different cell types and individuals. Additionally, the creation of synthetic SHSs, strategically inserted into well-characterized genomic regions, could be a valid strategy to avoid transgene silencing and mitigate unpredictable epigenetic effects, enabling more consistent and durable gene expression. Moreover, innovations in precision editing, such as high-fidelity Cas9 variants and prime editing, can mitigate off-target activity, while transient inhibition of non-homologous end joining could enhance homology-directed repair and thereby improve targeted integration efficiency. Advances in nanoparticle-based delivery systems may further reduce toxicity and facilitate the insertion of large genetic payloads in therapeutic contexts. Additionally, the increasing availability of population-scale genomic datasets could support the development of personalized SHS-insertion strategies. Tailoring the insertion to an individual’s genomic and epigenetic landscape can improve both safety and efficacy, particularly in patient-specific approaches such as iPSC-based regenerative therapies. Finally, ethical oversight must evolve in parallel with technical capabilities. Robust global guidelines and transparent public discourse are essential to ensure informed consent, protect genetic privacy, and promote equitable access while guarding against misuse. This is particularly important as germline editing becomes technically more feasible and socially contentious. In summary, the use of SHSs holds exceptional promise for the future of genetic engineering, functional genomics, and gene-based therapies. However, realizing their full potential will require overcoming considerable scientific, technological, and ethical barriers. With continued innovation, rigorous validation, and responsible governance, SHSs could become foundational elements of precise, safe, and effective genome engineering in both research and clinical settings.

## Data Availability

No new data were created or analyzed in this study. Data sharing is not applicable to this article.
